# Characterization of Co-Cr-W Dental Alloys with Veneering Materials Manufactured via Subtractive Milling and Additive Manufacturing LDED Methods

**DOI:** 10.3390/ma15134624

**Published:** 2022-06-30

**Authors:** Óscar Barro, Felipe Arias-González, Fernando Lusquiños, Rafael Comesaña, Jesús del Val, Antonio Riveiro, Aida Badaoui, Félix Gómez-Baño, Juan Pou

**Affiliations:** 1 LaserOn Research Group, School of Engineering, University of Vigo, Lagoas-Marcosende, E-36310 Vigo, Spain; obarro@uvigo.es (Ó.B.); felipeag@uvigo.es (F.A.-G.); flusqui@uvigo.es (F.L.); racomesana@uvigo.es (R.C.); jesusdv@uvigo.es (J.d.V.); ariveiro@uvigo.es (A.R.); 2 Corus-Fegoba, C/San Jorge 18, E-15002 A Coruña, Spain; felix.gomez@corusdental.com; 3 Galicia Sur Health Research Institute (IIS Galicia Sur), SERGAS-UVIGO, E-36310 Vigo, Spain; 4 Materials Engineering, Applied Mechanics and Construction Department, University of Vigo, EEI, Lagoas-Marcosende, E-36310 Vigo, Spain; aida@uvigo.es

**Keywords:** Co-Cr alloy, additive manufacturing, laser-directed energy deposition (LDED), dental restorations, adhesion, microstructure, milling

## Abstract

Laser-directed energy deposition (LDED) is an additive manufacturing (AM) technology which can be an alternative to the traditional subtractive milling process for the obtention of porcelain-fused-to-metal (PFM) prosthesis. Still, the adhesion performance of the veneering ceramics for this material has been not studied yet. The main objective of this study is to perform a systematic comparison of the adhesion performance of Co-Cr-W metal frameworks obtained through LDED and conventional milling techniques. Comparison includes microstructural, superficial, and adhesion analysis. Co-Cr manufactured via LDED technique presents similar behavior (*p* < 0.05) in comparison to the material obtained via milling techniques, and its performance was validated with the veneering ceramics and veneering composites currently employed in the dental industry.

## 1. Introduction

Teeth are vital organs in vertebrates whose main function is chewing to optimize the reaction of digestive enzymes with food [1]. In humans, the function of the teeth is not limited to the masticatory function; they also have a fundamental role in speech as well as an aesthetic purpose. Therefore, the loss of one or several of these organs, known as edentulism, can affect the overall performance of the patient as well as their psychological health. Teeth suffer from high mechanical stress due to the masticatory process. It is estimated that the enamel and the dentin endure around 3000 cycles of 20 MPa of pressure each day [2]. In case of missing teeth, apart from the aesthetic effect, the force exerted by the mandibular bones is divided among fewer teeth, increasing the stress on them and increasing the risk of losing more dental units. Therefore, the restoration of these lost units is highly recommended.

Currently, the restoration of lost teeth (or teeth in critical condition) by means of fixed dental prostheses is considered the most appropriate therapeutic procedure because it allows a better distribution of occlusal loads during the mastication process [3,4] and thus increases the durability of the remaining natural teeth [5].

Among the different types of fixed dental prostheses, there is a group that is characterized by having a high quality: porcelain-fused-to-metal prostheses (PFM). These prostheses are characterized by having an internal metal structure covered with multiple layers of ceramics in the crown part. This mix combines the advantages of ceramics, such as their high hardness and aesthetic appeal similar to that of the enamel of the teeth, with the advantages of metals, such as their high toughness and high resistance to fatigue. Therefore, these prostheses are of great interest for both patients and the dental prosthetic industry, currently being one of the most employed solutions for edentulism.

PFM dental prostheses are positioned over dental implants inside the patient’s oral cavity. Due to being inside the buccal environment, the prosthesis must comply with certain health security standards. As an example, the metal of the structure must possess adequate mechanical performance and be compatible with veneering ceramic materials. That is why Co-Cr are the most used alloys for this application, possessing all of these properties: biocompatibility [6], corrosion resistance [7], and compatibility with the veneering ceramics [8,9]. Among the different types of ceramic coatings available for dental PFM restorations, the most used for their excellent mechanical, functional, and aesthetic properties are feldspathic coatings. These porcelains are formulated in a very precise way to achieve these wear resistance and aesthetic properties and are typically composed of a crystalline phase (leucite) dispersed in an amorphous matrix (glass). Their chemical composition typically includes silicon dioxide (SiO_2_), aluminum oxide (Al_2_O_3_), sodium oxide (Na_2_O), and potassium oxide (K_2_O) [10].

An alternative type of coating to ceramics is resin composites [11,12]. Indirect-light-cured resin composites have been used in dental restorations due to their acceptable aesthetics, their wear resistance similar to that of dental enamel, and their ease of manipulation [13,14,15]. These veneer materials are softer than ceramic ones [16], and they are currently employed when a softer coating is required. This is the case of patients in whom all the teeth have been replaced by dental prostheses, both in the upper and lower jaw. In this case, the application of a ceramic coating on the upper prosthesis and a composite-based coating on the lower one is indicated, so that the bite is carried out smoothly.

**Figure 1:**
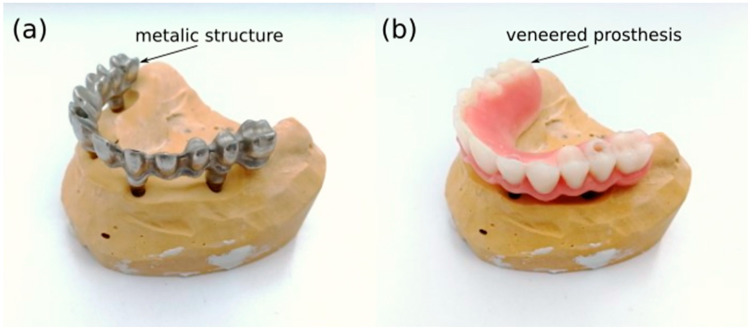
Dental prosthesis after metallic structure manufacturing (**a**) and in final form after veneering processes (**b**).

The manufacturing process of the PFM dental prostheses, once the Computer Aided Design (CAD) model is generated, comprises two steps: metallic structure manufacturing (Figure 1a) and veneering process (Figure 1b). For the manufacturing of the metallic structure, the most employed technique has traditionally been the casting through lost wax method [17]. However, this technique has been superseded by the milling technique. The reason for this change is that the milling technique has allowed an increase in the precision of final parts and has notably increased the certainty of producing a consistent homogeneous inner material [18].

However, Co-Cr alloys are expensive, and the milling from disc technique has a low material efficiency for personalized parts, such as dental prostheses. That is why in recent years, additive manufacturing (AM) techniques have been studied as an alternative to traditional milling for Co-Cr alloys [19,20,21,22].

**Figure 2:**
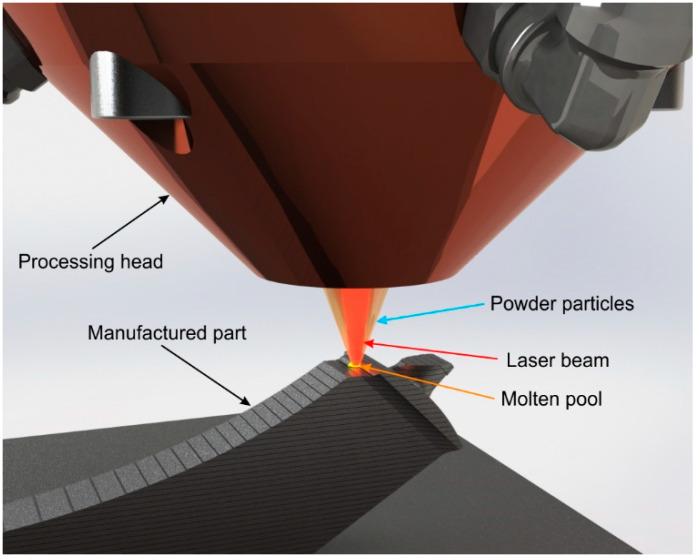
Representative model of the LDED (laser-directed energy deposition) technique with coaxial particle injection setup.

One of these AM techniques is laser-directed energy deposition (LDED, Figure 2). This technique consists of a laser beam that melts a substrate. Through the supply in real time of material (usually a powder stream) to the processing spot, a clad track is formed. Through the stacking of multiple layers of clad tracks following a given three-dimensional CAD model, the final part is generated [23].

The LDED technique is characterized by the generation of a high-quality metallurgical bonding between layers as well as by its provision of high material deposition rates [24,25]. Additionally, the microstructure can be tuned through the modification of processing parameters [26]. This is of great importance in Co-Cr-W alloys because the microstructure modification will alter notably their mechanical performance [27]. LDED can be employed with a wide range of different materials [28,29,30,31,32,33]. In the dental prosthetic field, it has been reported that LDED can generate a Co-Cr-W material that complains with the mechanical and electrochemical requirements of 22674 ISO standards [34]. However, since this metal must work with dental veneering ceramics, an important part of its performance is related to the overall performance of the metal–ceramic system. The characteristics of the commercial veneering ceramics are optimized to the materials currently employed in the dental industry. The materials generated through LDED present different microstructures than the commercial ones [24], so these ceramics may not work correctly with them. Studies of the adhesion of veneering ceramics have been performed for materials generated by other AM technologies in the dental field, such as selective laser melting [35]. However, there is a lack of similar studies for LDED-generated materials [36]. 

The main objective of this work was to assess the adhesion of commercial veneering ceramics to Co-Cr-W alloy produced by LDED. Adhesion of a veneering resin composite to the LDED-generated Co-Cr-W alloy was also studied. Additionally, these results are compared with the results obtained with a Co-Cr-W alloy machined through the milling from disc technique in order to have a reference of adhesion performance.

## 2. Materials and Methods

### 2.1. Metallic Specimen Generation

Two different manufacturing techniques (milling from disc and LDED) were employed to manufacture the metallic specimens using Co-Cr-W dental alloys. The composition of the alloys used in the present study is collated in Table 1.

**Figure 3:**
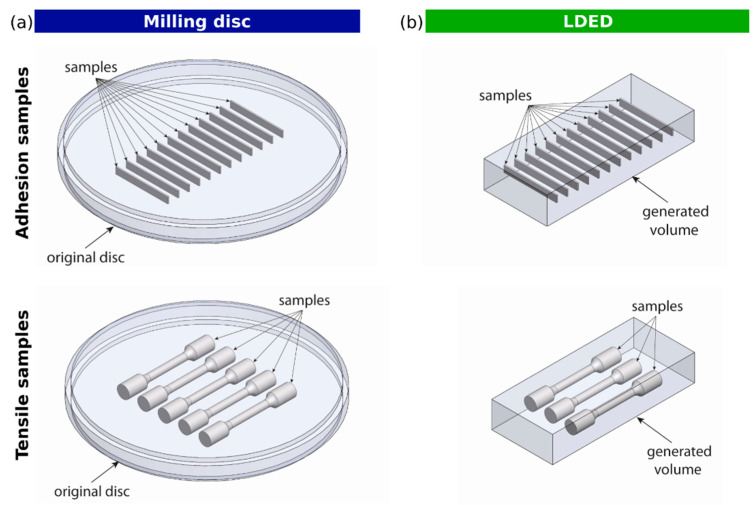
Representative model of the adhesion and tensile specimens’ positions inside the disc (**a**) and LDED (**b**) materials.

Milling specimens were machined from commercial Co-Cr-W Kera^®^-Disc dental discs using a CAD/CAM milling machine (DT2, Dyamach Italia, Mussolente, Italy) that employed solid carbide cutting tools. The position of the specimens and its orientation inside the bulk material is shown in Figure 3a.

LDED specimens were generated using a proprietary LDED additive manufacturing station (LaserOn Research group, Vigo, Spain). This system is composed of a 1600 W high-power diode laser power source employed to generate the molten pool in a Co-Cr building plate, a pneumatic powder feeder (Oerlikon Metco Europe GmbH, Raunheim, Germany), a coaxial laser head, and a computer numerical control (CNC) 3-axis positioning system enclosed in an argon-controlled ambient inert atmosphere. Commercial Co-Cr-W powder (Starbond Easy Powder 30+) with a particle size between 10 and 70 µm was used as precursor material. The near-net-shaped specimens generated were set apart from the building plates and machined in a milling CAD/CAM system (DT2, Dyamach Italia, Mussolente, Italy). Metal specimens of each technique (milling and LDED) were generated in a parallelepiped shape with 25 (±1) mm × 3.0 (±0.1) mm × 0.5 (±0.05) mm total dimensions, according to ISO 9693 [37] specifications. In order to ensure the thickness value (tolerance of ±0.05), all specimens were milled and ground with 320-grit grinding paper until achieving final dimensions with no additional thermal treatments. The metal substrates were divided into 2 groups (*n* = 12 for each group)—one group for feldspathic ceramic veneering and the second group for composite veneering. The representative position of the specimens and its orientation inside the bulk material is shown in Figure 3.

### 2.2. Veneering Materials

Two types of veneering materials were used in this study: a feldspathic ceramic and a resin matrix composite.

**Figure 4:**
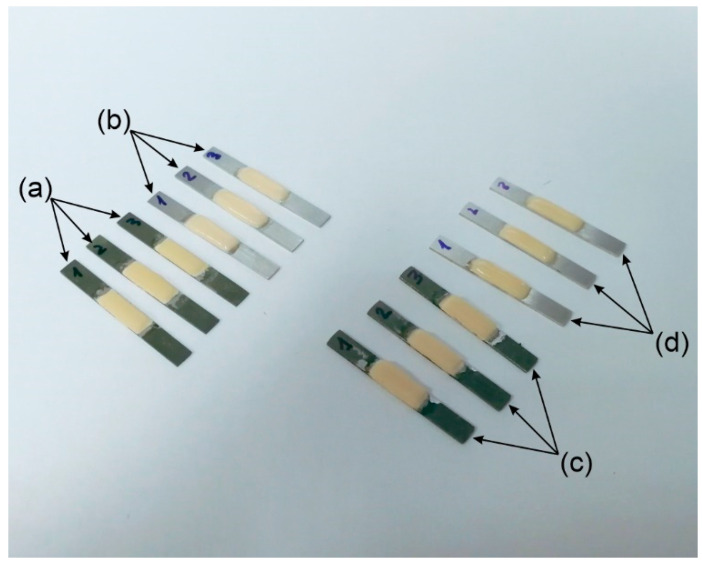
Sample of adhesion specimens of ceramic-veneered (**a**,**c**) and composite-veneered (**b**,**d**) milling disc (**a**,**b**) and LDED (**c**,**d**).

The procedure to apply the ceramic veneer was the following. First, the surface of the metallic substrates, where the ceramic would be placed, was blasted with aluminum oxide (Al_2_O_3_, 100 μm particles). A bonding agent (BONDING, NOBIL METAL S.P.A., Villafranca d’Asti, Italy) was applied in the deposition zone. Then, the substrates were furnace-cured following the procedure reported in Table 2. Second, feldspathic dental porcelains (GC Initial MC, GC Europe, Lovaina, Belgium) were applied in layers (opaque, dentin, enamel) according to ISO 9693, with dimensions of 8.0 (±0.1) mm × 3.0 (±0.1) mm × 1.1 (±0.1) mm in the center of the sample (Figure 4a,c). Ceramic layers were applied employing an aqueous suspension of the ceramic powders with the help of a paintbrush, followed by the corresponding furnace cycles under vacuum. Table 2 reports the firing temperatures and times used for the ceramic.

For the composite veneering process, microhybrid resin composite (Gradia, GC Europe, Lovaina, Belgium) was applied according to ISO 9693, with dimensions of 8.0 (±0.1) mm × 3.0 (±0.1) mm × 1.1 (±0.1) mm (Figure 4b,d). Layers were applied employing a paintbrush and cured following manufacturer instructions.

### 2.3. Microstructural Characterization

#### 2.3.1. SEM-EDS Analysis

Materials generated by each technique (milling and LDED) were examined using a scanning electron microscope (SEM, Philips XL30, FEI Technologies Inc., Hillsboro, OR, USA) coupled with an energy-dispersive X-ray spectroscopy (EDS) unit (EDAX PV9760, EDAX Inc., Mahwah, NJ, USA). Backscattered electron detection (BSE) was employed for imaging and together with EDS for microstructural analysis. Samples were cut in half, embedded in conductive resin, and ground and polished using colloidal silica (0.04 μm). An accelerating voltage of 20 kV and 10 mm of working distance were employed as main SEM parameters.

#### 2.3.2. XRD Analysis

One metallic specimen of each technique (milling and LDED), four in total (both before and after veneering firing cycles) were analyzed using X-ray diffraction technique (XRD, Siemens D5000, KS Analytical Systems, Aubrey, TX, USA) over the 30–120° 2θ range (40 kV, 30 mA, 0.05° step size, 3 s step time) with monochromated Cu-Kα radiation (λ = 1.54 Å). Samples (8 mm × 8 mm × 5 mm) were cut in half and ground using 320-grit SiC paper in order to compare the crystal structure of the metallic materials in samples with similar size to the real dental restorations.

### 2.4. Mechanical Analysis

The Vickers microhardness was measured in accordance with the ISO 6507-1:2018 standard. One specimen (8 mm × 8 mm × 8 mm) generated by each process (disc milling and LDED) was tested using a microindenter (HMV-G, Shimadzu Corporation, Kyoto, Japan), which applied a load of 9.81 N for 10 s of dwell time. The test was repeated in 5 different zones and averaged in order to achieve a representative measurement.

**Figure 5:**
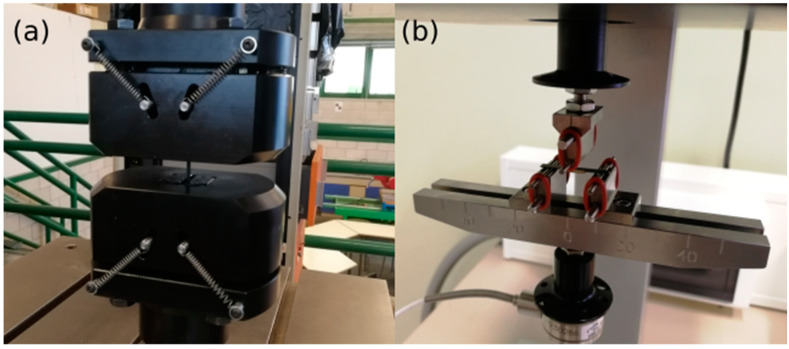
Representative image of the adhesion (**a**) and tensile (**b**) tests performed in the current study.

Tensile mechanical tests were performed in accordance with the ISO 22674:2016 standard, making the length of the samples of 42 mm with 3 mm of diameter in the evaluation zone. For the tensile test, 24 samples generated by each technique (disc milling and LDED with and without furnace veneering cycles) were tested. The tensile tests were performed using a tensile test machine (Figure 5a) LFV 25, Walter + Bai AG, Löhningen, Switzerland), setting the stroke motion at 1.5 mm/min until fracture. During this process, an extensometer (3542-010M-020-ST, Epsilon Technology Corp, Jackson, WY, USA) was used to determine the elastic modulus of the materials.

### 2.5. Surface Analysis

Profilometry analyses of the sample’s surfaces were performed to assess surface roughness before veneering processes. Surface-based analyses were performed employing a white light interferometric optical profilometer (Profilm 3D, Filmetrics, KLA Corporation, Milpitas, CA, USA). Surface-based roughness values were obtained via the arithmetic mean height of the surface Sa (μm) and via the ratio of increment in the interfacial area to the area of a perfectly flat surface Sdr (%), according to the ISO 25178 standard. The test was repeated for 3 different samples of each type and averaged in order to achieve a representative value.

Wettability at room temperature was determined using a monochromatic goniometer measuring system (Ossila Ltd., Sheffield, UK) and with deionized water as test fluid. Contact angle measurements were performed using the sessile drop method with 2.5 µL drops of deionized water deposited on the samples with a micropipette, according to the EN 828:2013 standard.

### 2.6. Veneering Coating Adhesion Analysis

The bond strength of the adhesion specimens was measured in 6 specimens generated by each technique, 24 in total (disc milling and LDED for feldspathic ceramic and composite coatings) using a 3-point bending test (Figure 5b, ElectroForce 5500, TA instruments, New Castle, DE, USA), according to the ISO 9693 standard, setting the head motion to 1.5 mm/min until failure of the joint.

Optical microscopy (SMZ1000, Nikon Metrology, Brighton, MI, USA) was used to analyze the failure mode of adhering coatings after debonding. The failure mode can be classified into three types: adhesive, where less than 20% of the area is covered with remaining ceramic in the sample surface; cohesive, where more than 80% of the area is covered; and mixed, being the situation intermediate between the other cases.

### 2.7. Statistical Analysis

All raw data were processed using R-project (v.4,12, R Foundation, Vienna, Austria) for data analysis and statistical computing of the tests results. Data from tensile mechanical tests were compared using two-way ANOVA followed by the Tukey HSD test (level of significance, alpha = 0.05). Data from adhesion tests were compared using two-sample Kolmogorov–Smirnov test significance (level of significance, alpha = 0.025).

## 3. Results and Discussion

To correctly evaluate the adhesion performance of the material generated by LDED following the international standards requirements, we have compared its performance with the material generated by the milling technique. In order to have a global overview of the materials adhesion performance, the analyses have been carried out in a multidisciplinary approach: from a microstructural analysis to an adhesion flexural test.

### 3.1. Microstructural Analysis of Co-Cr-W Alloys

**Figure 6:**
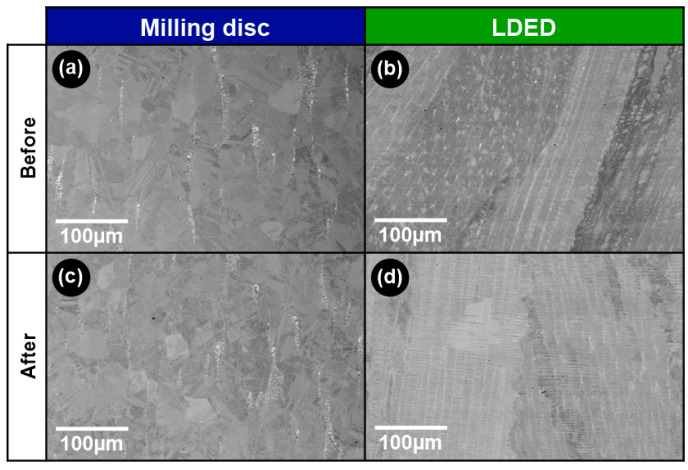
SEM micrographs showing a longitudinal section of samples generated by each technique: disc milling (**a**,**c**) and LDED (**b**,**d**) before (**a**,**b**) and after (**c**,**d**) veneering furnace cycles.

Figure 6 shows the microstructure of the Co-Cr-W alloys obtained by means of the two techniques (milling and LDED), before and after being subjected to the ceramic veneering furnace cycles. The CAD/CAM milling disc material is composed by a homogeneous structure of equiaxed grains with intergranular segregation (Figure 6a). This microstructure is characteristic of the continuous casting or hot rolling processes [38], techniques employed as manufacturing methods in dental discs [39]. It can be observed that there are segregation patterns aligned with the axial disc direction, an effect caused by the unidirectionality of the thermal gradients during the manufacturing method of the disc. Interdendritic segregation in these Co-Cr-W alloys can be identified as σ and laves phases [40], and their size is related to reduced cooling rates that promote their segregation. After the furnace processes of veneering (Figure 6c), the disc material did not show a remarkable change in the size of the segregation zones or grain size.

On the other hand, material obtained through the LDED process showed a columnar grain structure aligned with the building direction. This column structure is composed of columnar dendrites with submicrometric interdendritic segregation aligned with the columnar grains (Figure 6b). This columnar growth is mainly caused by the unidirectional thermal gradient and the remelting of previous layers during the manufacturing process, which produces the grain continuity among them [41]. This preferred orientation is a characteristic of LDED process that in some cases even leads to epitaxial grain growth [42,43] In the LDED material, similar to the disc material, the furnace cycles of veneering do not notably modify the microstructure (Figure 6d).

**Figure 7:**
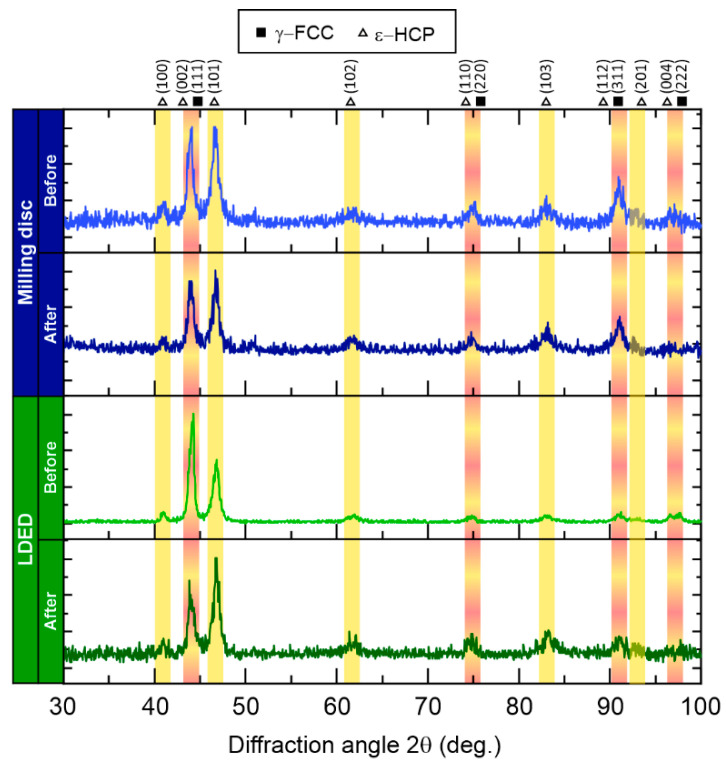
X-ray diffraction patterns of both milling disc and LDED techniques before and after furnace veneering cycles. γ-FCC (square symbol, red band) and ε-HCP (triangle symbol, yellow band) phases’ peak angles are highlighted.

To identify the phase composition of both materials, XRD analyses (Figure 7) were conducted. Both materials are composed of stable γ-FCC and metastable ε-HCP phases with similar diffraction patterns. This phase structure is in agreement with that found in previous works of Co-Cr dental alloys [44,45,46]. Additionally, there is not a significative change in phase stability after the thermal veneering cycles, in concordance with SEM analysis.

### 3.2. Mechanical Properties of Co-Cr-W Alloys

In a previous study, we assessed that both materials obtained through milling disc and LDED techniques using the same alloys possess very similar behavior in terms of yield strength, ultimate tensile strength, elongation after fracture, and toughness, with no significant differences between techniques (*p* > 0.05) [34]. Both materials are compliant with ISO 22674 (yield strength > 360 MPa and elongation > 2%). However, properties that directly affect the surface and adhesion tests, such as hardness or Young modulus, had to be addressed in the present study. Therefore, in the present study, both properties have been assessed through mechanical analysis following the standards ISO 6507-1 for hardness and ISO 22764 for Young modulus.

Substrate hardness is an important property that influences coating–substrate adhesion. It has been demonstrated that increasing substrate hardness implies improving adhesion of coatings to substrates, especially on hard coatings, such as ceramics [47]. Material obtained from disc milling possessed a significant (*p* < 0.05) lower microhardness of 272 (SD: 19) HV1 than LDED material with 303 (SD: 11) HV1, with an 11% increase of the latter but with high variability. This difference is also shown after the furnace cycles of veneering, with an increase of 17% for the LDED material (milling disc: 273 (SD: 17), LDED: 320 (SD: 9)). Finally, Young moduli of both materials have been obtained for use in bonding strength analyses. Milling disc material possessed 240 (SD: 7) MPa, whereas LDED samples had 238 (SD: 6) MPa of elastic modulus; both materials possessed similar behavior with no significative differences.

### 3.3. Surface Roughness and Wettability of Co-Cr-W Alloys

**Figure 8:**
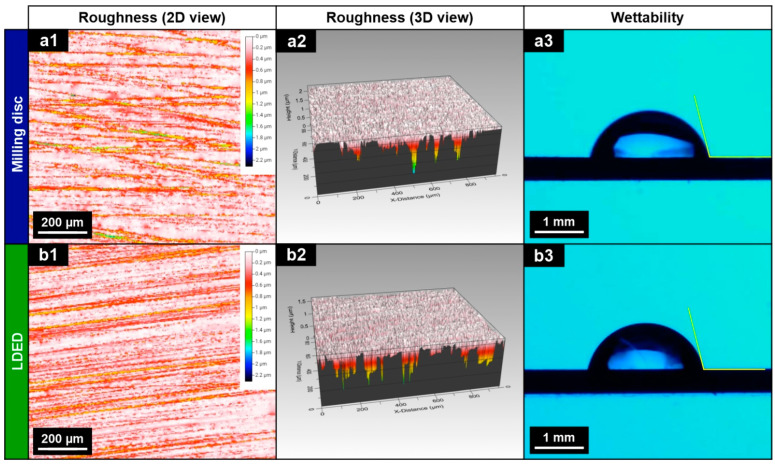
Summary of surface characteristics of the materials before veneering processes: 2D surface view (**a1**,**b1**), 3D surface view (**a2**,**b2**) and contact angle (**a3**,**b3**) tests for disc milling (**a1**–**a3**) and LDED (**b1**–**b3**) techniques.

Since there is a significant increase in hardness of the LDED material, additional surface analyses have been performed. To quantitatively evaluate the surfaces obtained before veneering processes and detect differences in roughness, all surfaces were measured employing optical profilometry. As can be seen in Figure 8 and Table 3, both materials possess quite similar surfaces with nearly the same Sa and Sq.

Sa and Sq standard roughness parameters are the usual parameters employed for identifying and measuring surfaces. However, the point of view of this study is more oriented toward evaluating the interaction of the contact surface with the liquified ceramic (the way it is deposited). In the case of contact surfaces, these Sa and Sq parameters are not sufficient to determine their tribological properties [48]. That is why Kurtosis and Skewness have been also determined. Both parameters provide a good correlation with the tribological properties. The Sku parameter shows that both materials are greater than 3, meaning that both materials possessed a distribution of high peaks with low valleys [49]. On the other hand, the Ssk parameter is negative in both materials, meaning that the symmetry of the mean line of both materials is oriented in the same direction and very similar. All roughness results between materials are very similar, suggesting that the surface performance will be also similar.

To verify the wettability of the ceramic during the deposition process, a wettability analysis was also performed (Figure 8a3,b3). Wetting angle results (Table 3) are in agreement with roughness results, being very similar for both properties, showing a behavior with no significant differences (*p* > 0.05) between the materials generated by the two techniques (milling and LDED).

### 3.4. Veneering Coating Adhesion to Co-Cr-W Alloys

In order to determine the bonding strength of the veneering coatings to the two materials generated by milling or by LDED techniques, ceramic-coated and composite-coated specimens were subjected to three-point bending test according to the ISO 9693 standard. The force was measured through the experiments and the bonding strength of the material (σ) was calculated following the equation

σ (Pa) = k (m^−2^) × F (N), (1)

where k is a calculated variable dependent on the Young modulus and the measured thickness of the metallic Co-Cr-W samples and F is the load that causes debonding between the metal part and the ceramic. The value of k has been determined following ISO 9693 for each sample using the Young modulus obtained in the mechanical analyses and the corresponding thickness of each sample.

**Figure 9:**
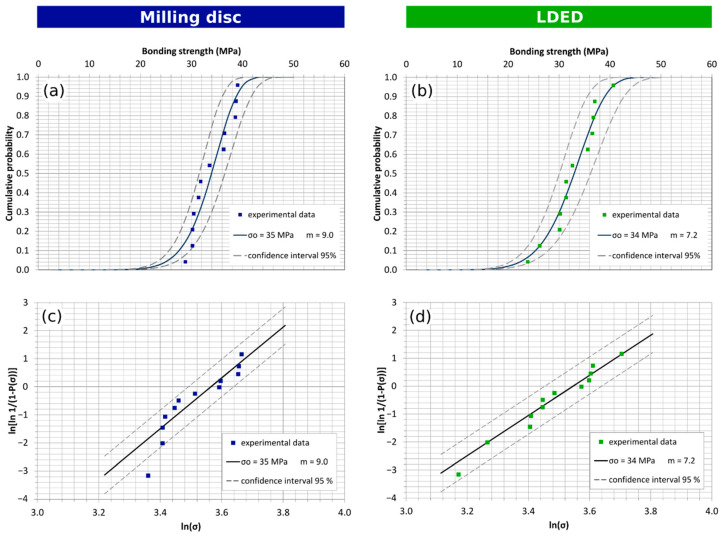
Bending strength test for ceramic-coated samples: Weibull plot and cumulative failure probability plot for (**a**,**c**) milling disc (Weibull characteristic strength σ_0_ = 35 MPa; Weibull modulus m_cor_ = 9.0); (**b**,**d**) LDED (Weibull characteristic strength σ_0_ = 34 MPa; Weibull modulus m_cor_ = 7.2).

The experimental cumulative failure probability and Weibull graph for the ceramic-coated specimens generated by milling from disc are shown in Figure 9a,c, respectively, and equivalent experimental charts for the ceramic-coated LDED specimens are shown in Figure 9b,d, respectively. The Weibull characteristic bonding strength of the ceramic-coated specimens reaches 35 and 34 MPa, for the disc machined and the LDED samples, respectively. The obtained corrected Weibull modulus is lower for the LDED material, with a value of 7.2, than for the machined material, with a value of 9.0.

These results show that the bonding strength of both materials (Disc: 35 MPa, LDED: 34 MPa) is nearly the same, indicating that the LDED material will perform similarly to the milled disc material with a slightly higher dispersion of the results. Both materials have a performance greater (Disc: 40%, LDED: 36%) than the minimum of 25 MPa set by the 9693 ISO standard.

**Figure 10:**
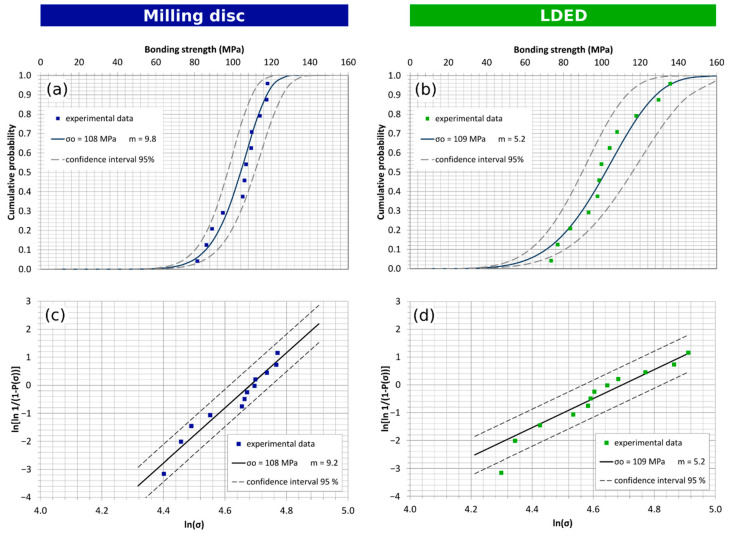
Bending strength test for composite-coated samples: Weibull plot and cumulative failure probability plot for (**a**,**c**) milling disc (Weibull characteristic strength σ_0_ = 108 MPa; Weibull modulus m_cor_ = 9.2); (**b**,**d**) LDED (Weibull characteristic strength σ_0_ = 109 MPa; Weibull modulus m_cor_ = 5.2).

The experimental cumulative failure probability and Weibull graph for the composite-coated disc-machined specimens are shown in Figure 10a,c, respectively, and equivalent experimental charts for the composite-coated LDED specimens are shown in Figure 10b,d, respectively. The Weibull characteristic bonding strength of the composite-coated specimens reaches 108 and 109 MPa, for the milling and the LDED samples, respectively. Again, both materials possess nearly the same Weibull bonding strength, being around 3.5 times that of the ceramic-coated specimens and 330% greater than the minimum of 25 MPa required by the 9693 ISO standard. The LDED specimen population showed higher dispersion, the corrected Weibull modulus for the LDED material has a value of 5.2, while the machined material has a Weibull modulus of 9.8. That result may be caused by the fact that all LDED materials broke by rupture of the composite material instead of adhesion fracture. ISO 9693 standard is defined for ceramic materials, and the composite ones perform three times better, so it is possible that the layer of composite is not enough for the tension stress applied to the deposited layer. This will require a higher section to avoid this fracture.

Despite of the slightly differently fitted Weibull modulus found for the ceramic-coated and composite-coated samples that could describe a higher dispersion of the results in the LDED case, the two-sample Kolmogorov–Smirnov test with a significance of α = 0.025 does not verify the hypothesis that milling and LDED materials are from different distributions. Therefore, the assumption that the bonding strength for the LDED specimens and the machined specimens follow the same distribution and possess same behavior with a significance of 0.025 is valid.

**Figure 11:**
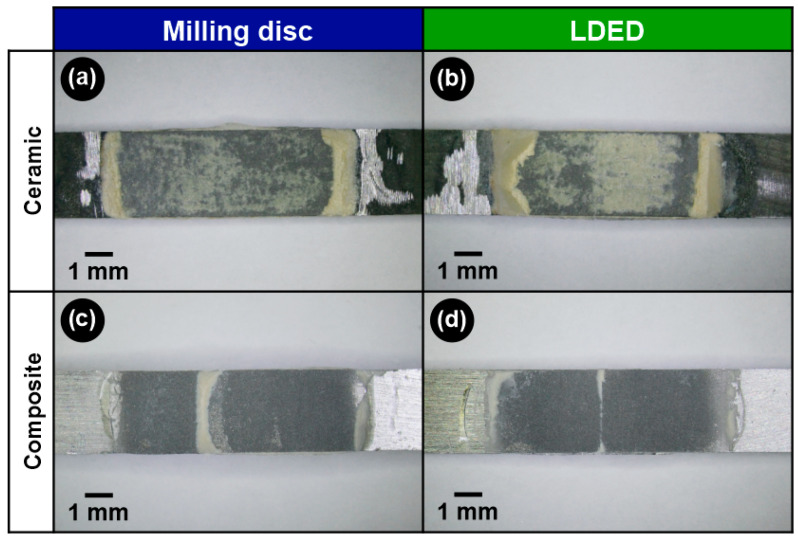
Optical microscopy images of surfaces after debonding for both milling disc (**a**,**c**) and LDED (**b**,**d**) materials using ceramic-veneering (**a**,**b**) and composite-veneering (**c**,**d**) techniques.

That conclusion is corroborated by optical microscopy, where debonded surfaces show a similar failure mode between techniques (Figure 11). Both milling disc (Figure 11a) and LDED (Figure 11b) ceramic groups showed a mixed–nearly cohesive failure mode with remnants of ceramic in the borders of the deposited coating. In the composite case, the groups showed a mainly adhesive failure mode (Figure 11c,d). Usually, adhesive failure is not the ideal situation due to the fact that this indicates a reduced bond adherence between the original metal and the coating [50]. However, in this case, the adhesion strength was three times greater than that of the ceramic coating, so their good performance is ensured.

## 4. Conclusions

Co-Cr-W dental alloys manufactured by a laser-directed energy deposition (LDED) technique show similar phase composition and wettability properties to commercial counterpart material obtained from disc milling. LDED material complied in adhesion behavior with veneering ceramics and composite materials following ISO 9693 standards.

The present work demonstrates that milling disc materials could be replaced by the LDED material with no modifications to ceramic veneering manufacturing supply. This fact provides an easier implementation in the manufacturing process of the porcelain fused to metal dental prostheses.

## Figures and Tables

**Table 1:** Chemical composition (wt. %) of the Co-Cr-W alloys used in this work.

Group	Name	Information	Co	Cr	W	Mo	Si	Mn	Fe	Manufacturer
Disc milling	Kera^®^-Disc	Manufacturer	Bal.	27.75	8.45	-	-	0.25	0.2	Eisenbacher Dentalwaren ED GmbH, Wörth am Main, Germany
		XRF	Bal.	24.1	8.59	<0.01	2.64	0.18	0.1	
LDED	Starbond Easy Powder 30+	Manufacturer	Bal.	27.5	8.5	-	1.6	<1	<1	Scheftner GmbH, Mainz, Germany
		XRF	Bal.	22.41	7.84	0.04	1.3	0.23	0.17	

Note: Manufacturer: Composition certified by the manufacturers. XRF: Composition obtained through X-ray fluorescence technique.

**Table 2:** Summary of the feldspathic veneering method parameters used in this study.

Process	Pre-Heating Temp. (°C)	Drying Time (min)	Heating Rate (°C/min)	Final Temp (°C)	Holding Time (min)	Vacuum
Bonding	650	6	55	980	1	Yes
Opaque	550	6	80	940	1	Yes
Dentin	580	6	55	900	1	Yes
Glaze	600	2	55	860	1	Yes

**Table 3:** Summary of the roughness parameters and angle of wettability obtained in the tested alloys, standard deviation (SD) in parentheses.

Material	Maximum Peak Height Sp (µm)	Maximum Pit Height Sv (µm)	Maximum Height Sz (µm)	Arithmetic Mean Height Sa (µm)	Root mean Square Height Sq (µm)	Skewness Ssk	Kurtosis Sku	Angle of Wettability (°)
Milling disc	0.24 (0.03)	1.97 (0.40)	2.21 (0.43)	0.17 (0.01)	0.23 (0.01)	−2.10 (0.11)	8.6 (1.0)	69.9 (6.9)
LDED	0.25 (0.03)	1.35 (0.15)	1.60 (0.17)	0.17 (0.01)	0.22 (0.01)	−1.57 (0.21)	5.6 (0.6)	74.3 (2.8)
